# New In Vitro Studies on the Bioprofile of *Genista tenera* Antihyperglycemic Extract

**DOI:** 10.1007/s13659-015-0077-z

**Published:** 2015-10-22

**Authors:** Daniela Batista, Pedro L. Falé, Maria L. Serralheiro, Maria E. Araújo, Paulo J. A. Madeira, Carlos Borges, Isabel Torgal, Margarida Goulart, Jorge Justino, Alice Martins, Amélia P. Rauter

**Affiliations:** Centro de Química e Bioquímica, Faculdade de Ciências, Universidade de Lisboa, Campo Grande, 1749-016 Lisbon, Portugal; Escola Superior Agrária de Santarém, Quinta do Galinheiro, 2001-904 Santarém, Portugal; Institute of Pharmaceutical Science, King’s College London, 150 Stamford Street, London, SE1 9NH UK

**Keywords:** *α*-Glucosidase inhibition, In vitro digestion, HPLC–DAD-ESI–MS/MS, Antioxidant activity, Flavonoid glycosides

## Abstract

**Abstract:**

The inhibition of *α*-glucosidase and glucose-6-phosphatase, two enzymes involved in the carbohydrate metabolism, is an important target to control glycaemia on individuals with type 2 diabetes. In this work we report for the first time the inhibition of both enzymes by the antihyperglycemic *n*-butanol extract from *Genista tenera* (Fabaceae). This extract decreased *α*-glucosidase and glucose-6-phosphatase activities to 0.97 and 80.25 %, respectively, being more effective than acarbose, and phlorizin, the positive controls, which reduced enzymes activities only to 17.39 and 96.06 %. Once inflammation and oxidative stress are related to diabetic impairments, the anti-inflammatory activity of the extract was also evaluated, through its inhibitory activity over COX-1 enzyme (47.5 % inhibition). Moreover, after induction of oxidative stress by UV radiation, the viability of irradiated rat liver hepatoma cells exposed to the extract was significantly higher (67.82 %) than that promoted by ascorbic acid, the positive control (45.05 %). In addition, the stability of the extract under gastrointestinal conditions was evaluated by HPLC–DAD-ESI–MS/MS. Flavonoid diglycosides were identified as the main constituents of the extract, and no alterations in the chemical composition nor in the antioxidant activity were observed after in vitro digestion with artificial gastric and pancreatic juices.

**Graphical Abstract:**

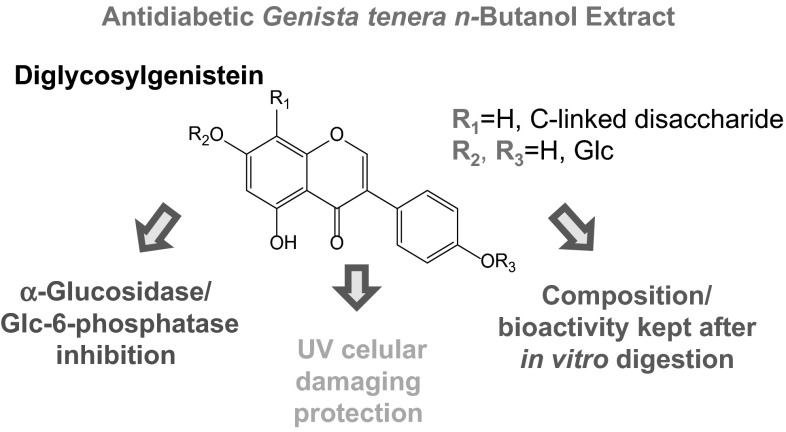

## Introduction

Type 2 diabetes is a major public health concern, due to the increasing incidence in developed and developing countries. The loss of patient’s quality of life, the economic burden associated with direct and indirect costs, the relevant morbidity, and increased mortality reinforce the needs to investigate new treatments and preventive options aiming to control diabetes. There is evidence that hyperglycaemia results in the generation of reactive oxygen species and a link between oxidative stress, inflammatory response and diabetes is now well established [1]. Antioxidants are known to exert beneficial effects in ameliorating the injurious effects of hyperglycaemia in different in vitro and in vivo models [2] and the intake of natural antioxidants, like polyphenols, is able to modulate a number of chronic inflammatory diseases including type 2 diabetes [3]. Amongst the phenolic compounds, flavonoids have been associated with beneficial effects reducing the risk of cancer, diabetes, cardiovascular and brain diseases. These effects are related to their radical scavenging effect along with other possible mechanisms such as anti-inflammatory properties [4]. Epidemiological, in vitro and in vivo studies support the beneficial effect of dietary flavonoids on glucose and lipid homeostasis and a review of the recent findings on the antidiabetic effects of dietary flavonoids, in particularly their cellular and molecular mechanisms of action, has been reported [5]. So, the screening of chemical candidates from herbal medicines is a promising approach for new drugs discovery to the prevention and treatment of diabetes and related complications [6]. Extracts and/or natural molecular entities can act on several mechanisms of type 2 diabetes and enzymes that regulate glucose metabolism are potential targets for controlling glucose balance and thereby blood glucose levels in diabetic patients [7]. A therapeutic approach to prevent hyperglycaemia is to retard the absorption of glucose through the inhibition of alpha-glucosidase. Inhibitors can prolong the overall carbohydrate digestion time and cause a reduction in the rate of glucose absorption, blunting the postprandial plasma glucose rise [8]. Glucose-6-phosphatase catalyses the final step of both hepatic gluconeogenesis and glycogenolysis and its inhibition also contributes to the reduction of endogenous glucose production [7].

*Genista tenera* (Fabaceae) is a medicinal plant used in the island of Madeira, Portugal, to control diabetes. Previous studies have shown that its *n*-butanol extract exhibits a significant antihyperglycemic activity on an animal model with diabetes induced by STZ, and no acute cytotoxicity or genotoxicity to human lymphocytes was observed in an in vitro assay [9]. Aiming to investigate the mechanism of action of this antidiabetic extract we report now the first studies concerning its inhibition of α-glucosidase and glucose-6-phosphatase enzymes. Since oxidative stress and inflammation are also related to diabetes impairments, the effect of the extract on cell viability after UV radiation injury and the inhibition of COX-1 enzyme were also evaluated. Finally, extract stability after in vitro digestion is also here reported for the first time.

## Results and Discussion

### Effect on Alpha-Glucosidase and Glucose 6-Phosphatase Inhibition

**Fig. 1:**
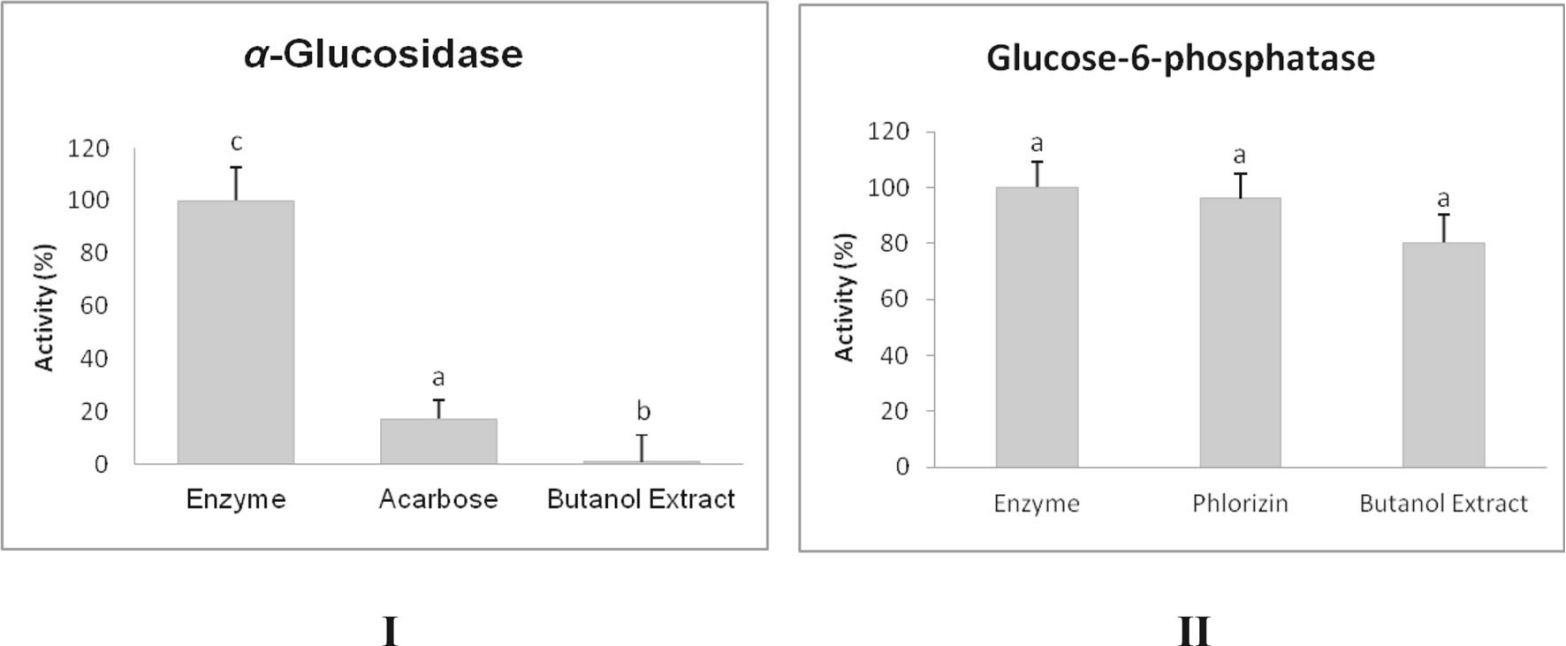
**I** Activity of rat small intestine *α*-glucosidase in the presence of *G. tenera n*-butanol extract. The activity was determined using maltose as substrate and acarbose as positive control. Each value is expressed as mean + standard deviation from a total of five replicates; **II** Activity of glucose-6-phosphatase from rabbit liver microsomes in the presence of *G. tenera n*-butanol, extract. The activity was determined using glucose-6-phosphate as substrate and phlorizin as positive control. Each value is expressed as mean + standard deviation from a total of 12 tests. For both assays the enzyme activity in the presence of the inhibitor is expressed in percentage of the enzyme activity without inhibitor (100 %). The same concentrations of extract, acarbose and phlorizin were used, 400 mg/L. Indexes (*a*–*c*) indicate statistically significant differences *P* < 0.05 analysed by the Tukey *a posteriori* test

The inhibitory activity of *G. tenera n*-butanol extracts on *α*-glucosidase and glucose-6-phosphatase was evaluated by spectrophotometry-based assays. The inhibition of *α*-glucosidase activity is of crucial importance in delaying absorption of dietary carbohydrates that contribute to postprandial hyperglycaemia in individuals with type 2 diabetes and is one important mechanism for the management of the diabetic condition [8, 10, 11]. The percentage activity of this enzyme, in the presence of *G. tenera n*-butanol extract, compared to the positive and negative controls, is depicted in Fig. 1I. The extract decreased enzyme activity to 0.97 % when compared to the commercial inhibitor acarbose, the positive control, which reduced enzyme activity only up to 17.39 %. These results suggest that inhibition of α-glucosidase plays an important role in the mechanism of the antihyperglycemic action of *G. tenera n*-butanol extract, thus contributing more effectively to the control of postprandial glycaemia in type 2 diabetes. Glucose-6-phosphatase is the enzyme that catalyses the final step of gluconeogenesis and glycogenolysis and its inhibition is also extremely important to control hyperglycaemia in diabetic patients [7, 12, 13]. The extract showed a tendency to inhibit this enzyme, whose activity decreased to 80.25 %, and revealed to be more effective than the positive control phlorizin, for which a remaining enzyme activity of 96.06 % was found, as shown in Fig. 1II.

Flavonoids are reported to inhibit alpha-glucosidase [8] and to alter hepatic gluconeogenic enzymes activity thereby reducing the endogenous glucose production [7], which suggests that the inhibitory activity of the *G. tenera n*-butanol extract can be attributed to its flavonoid composition.

### Anti-inflammatory Activity

It is well established that inflammation is also involved in diabetic impairments. In this context, a preliminary evaluation of the anti-inflammatory activity of *G. tenera* butanol extract was assessed by an in vitro assay, involving the inhibition of cyclooxygenase (COX, or Prostaglandin-H synthase, PGHS), a key enzyme in the synthesis of prostaglandin H2, which is a precursor for the biosynthesis of prostaglandins (PGs), thromboxanes, and prostacyclins. PGs support the release of further mediators of inflammation and cause the typical symptoms at inflammation sites. Therefore, PGHS has been regarded for a long time as an important target of most non-steroidal anti-inflammatory drugs (NSAIDs). We found that the *n*-butanol extract inhibited COX-1 enzyme (47.5 % inhibition at 0.5 mg/mL), and presented an IC_50_ = 291.60 μg/mL, which was compared with indomethacin (IC_50_ = 60 μg/mL), a widely used NSAIDs.

### Effect on Cell Viability

The influence of the extract on cell viability was also studied using the MTT method. The ability of cells to reduce MTT gives an indication of its integrity and metabolic activity, which in turn may be interpreted as a measure of cell viability. We studied the effect of *G. tenera n*-butanol extract on rat liver hepatoma cells (H-4-II-E), non-irradiated, and irradiated with UV-C radiation. The extract seems to have a positive effect on non-irradiated cells growth, once no statistically significant differences were observed when compared to ascorbic acid, the positive control. The viability of irradiated cells exposed to *G. tenera n*-butanol extract was significantly higher (67.82 ± 22.36 %) than that of non-treated cells (38.18 ± 7.73 %) and that promoted by the ascorbic acid (45.05 ± 26.35 %). So, the present study adds further support to the antioxidant properties of *G. tenera* extracts [9] and unveils new potential applications of the plant in long term protection against UV-related cellular damaging.

### Chemical and Biological Stability After In Vitro Digestion

**Fig. 2:**
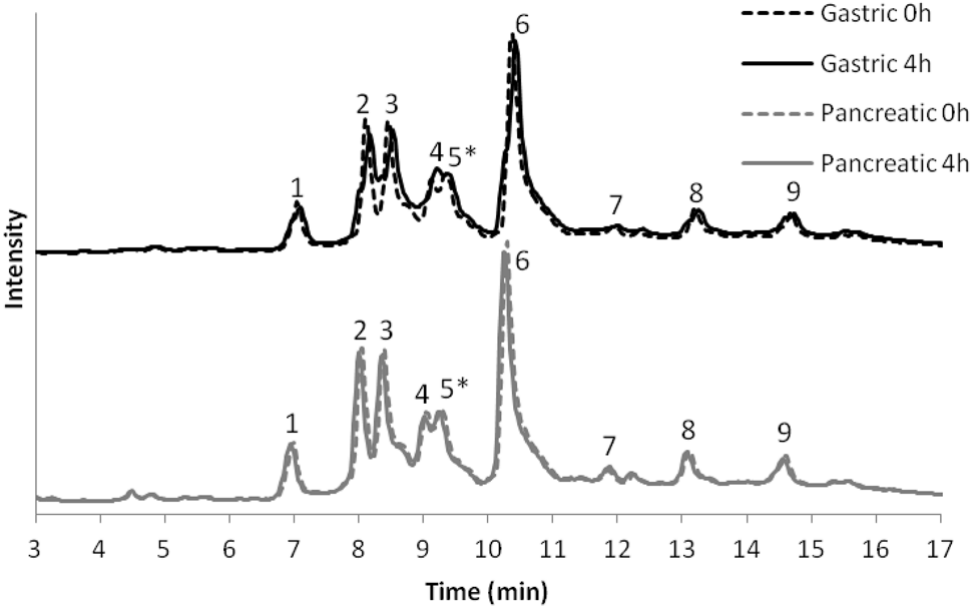
In vitro gastric and pancreatic digestions of *G.tenera* *n*-butanol extract analysed by HPLC–DAD:7-*O*-glucosyl-8-glucosylgenistein **(1)**, 8-(glucosyloxyglucosyl)genistein **(2)**, 4´,7-di-*O*-glucosylgenistein **(3)**, 6,8-diglucosylapigenin **(4)**, unknown (5*), 8-[apiosyl-(1 → 6)-glucosyl]genistein **(6)**, 3′,7-di-*O*-glucosylluteolin **(7)**, rutin **(8)**, and 3-*O*-rutinosylisorhamnetin **(9)**

In view of a potential use of *G. tenera* extract as a source of nutraceuticals, its stability under gastrointestinal conditions was analysed by HPLC. The extract was submitted to an in vitro digestion with artificial gastric and pancreatic juices and none of its constituents suffered hydrolysis (Fig. 2). Furthermore, the antioxidant activity was monitored throughout the digestion by the DPPH method. The IC_50_ value for DPPH extinction was 214.2 ± 0.3 µg/mL and, after 4 h digestion with gastric and pancreatic juices, this activity was 101.4 ± 10.7 and 112.1 ± 13.9 % of the initial, respectively. Hence, this extract may pass through the gastrointestinal tract keeping its composition, antioxidant capacity and therefore its biological properties.

### Chemical Analysis

**Fig. 3:**
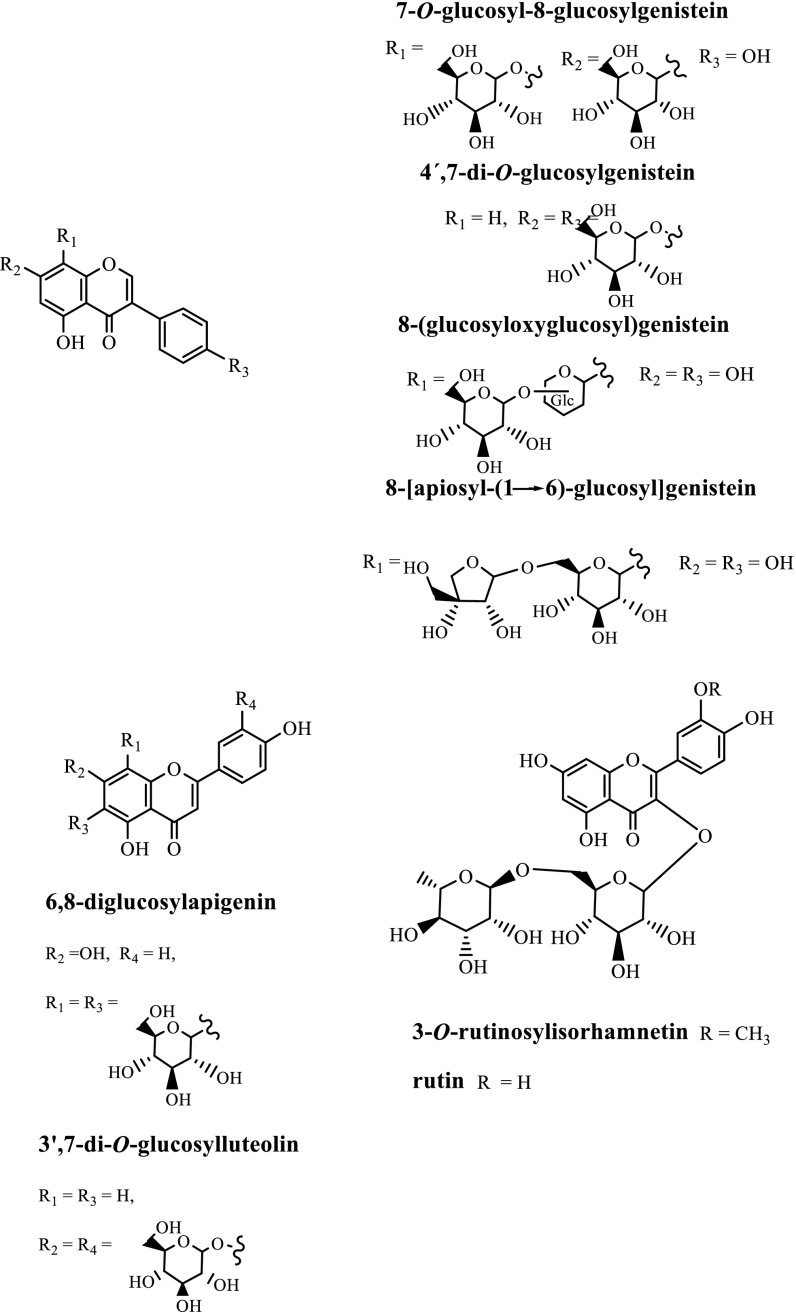
Structures of the flavonoids identified in the *n*-butanol extract of *G. tenera*

Aiming to identify the phytochemical composition of the extract, HPLC–DAD and HPLC–ESI–MS/MS analyses were performed. Nine peaks were detected (Fig. 2), before and after in vitro digestion, and chromatograms showed the same flavonoid profile, which structures are depicted in Fig. 3. The tentative identification of each peak (Table 1) was based on the fragmentation patterns in MS^2^ and MS^3^ experiments, UV spectra, and comparison with literature and MassBank database [14]. The MS^2^ spectrum of the deprotonated molecule of compound **1** at *m/z* 593 presents losses of 120 Da (^0,2^X^−^) and 90 Da (^0,3^X^−^) characteristic of a *C*-glucosyl flavonoid [15], affording the fragment ions at *m/z* 473 and 503, respectively. Waridel et al. [16] reported that the low abundance of the ^0,3^X^−^ fragment can be correlated with the glycosylation position, namely with glycosylation at C-8, suggesting that the relatively low abundance of ^0,3^X^−^ found for compound **1** indicates glycosylation at the C-8 position. From the DAD data, it can be inferred that the aglycone is an isoflavone, with the characteristic maximum wavelength at 261 nm and a shoulder at 295 nm, while flavones present also an intense band at 330–365 nm. Furthermore, the MS^3^ spectrum of the ion at *m/z* 473 shows a loss of 162 Da, a glucosyl moiety, which is indicative of an *O*-glucoside, suggesting that compound **1** is 7-*O*-glucosyl-8-glucosylgenistein. The deprotonated molecule of compound **2**, *m/z* 593 loses 252 Da resulting from the combined loss of glucosyl moiety and one ^0,3^X fragment and 282 Da, attributed to a combined loss of glucosyl moiety and ^0,2^X fragment, affording the ions at *m/z* 341 and 311, respectively, the latter with 100 % intensity. This behaviour suggests that a glucosylglucoside moiety is C–C linked to genistein, in accordance also with the observed UV absorption maxima. The loss of 28 Da (CO) observed in MS^3^ may result from contraction of ring C, which is commonly detected in isoflavones [17, 18]. Two consecutive losses of 162 Da indicate that compound **3** is a flavonoid diglucoside, showing an Y_1_^−^ ion at *m/z* 431. However, this compound ionizes as [M + HCOO]^−^ turning its assignment more difficult. Nevertheless, the fragmentation pattern is consistent with 4´,7-di-*O*-glucosylgenistein. The formation of [M + HCOO]^−^ although uncommon, has been reported in the literature for several compounds [19–21]. The DAD data suggest that compound **4** could be either a flavone or a flavonol since it exhibits an intense band II (314 nm). In mass spectrometry, *C*-glycosyl flavones suffer cross-ring cleavages of sugar residues yielding ions produced by losses of 90 and 120 Da, thus allowing differentiation from *O*-glycosyl flavones with losses of 162 Da for hexose, 146 Da for rhamnose and 132 Da for pentose moieties respectively [22]. The MS^2^ spectrum of the deprotonated molecule of compound **4**, *m/z* 593, presents the characteristic losses of a C-glucosyl flavonoid, i.e. the loss of 90 Da affording a ^0,3^X^−^ ion (*m/z* 503) and 120 Da affording a ^0,2^X^−^ ion (*m/z* 473). The MS^3^ of the ion at *m/z* 473 presents similar losses (90 and 120 Da), which can be indicative of a di-C-glucosyl flavonoid. A literature survey enabled us to propose compound **4** to be 6,8-diglucosylapigenin, since there is a close agreement between the data available in the literature [22, 23] with data acquired by us. The DAD data for compound **6** suggests that it is an isoflavone. The loss of 252 Da suggests the presence of a C–C linked apiosylglucosyl moiety, affording the ion ^0,2^X^−^ ion at *m/z* 311 bearing the apiosyl group. The characteristic loss of 222 Da is also detected by the presence of the ^0,3^X^−^ ion also bearing the apiosyl group at *m/z* 341. The MS^3^ of the ion at *m/z* 311 shows the loss of 28 Da, characteristic of an isoflavone. Interestingly, it was found that *retro*-Diels–Alder fragments, that are observed in flavones, flavanones, flavonols and chalcones were not found in the isoflavones studied, corroborating the results reported by Wang et al. [24] and references cited therein. Hence, the analysis of the data obtained for compound **6** suggests the structure of 8-[apiosyl-(1 → 6)-glucosyl] genistein previously assigned by Peng et al. [25]. The fragments obtained for compounds **7** (3′, 7-di-*O*-glucosylluteolin), **8** (rutin), and **9** (3-*O*-rutinosylisorhamnetin) were identified based on MassBank scores and records (Table 1).

**Table 1:** Putative identification of glycosyl flavonoids from *Genista tenera n*-butanol extract, by HPLC–DAD and HPLC-ESI–MS^n^

Peak	Rt (min)	λ_max_ (nm)	[M-H]^−^	*m/z* Product ions ESI–MS^n^ (relative abundance, %)	Identification (Massbank score and record)
**1**	7.1	261, 295 sh	593	[MS^2^ 593]: 503 (2), 473 (100) [MS^3^ 473]: 473 (100), 445 (24), 383 (2), 353 (2), 311 (12) 310 (25), 282 (12)	7-*O*-Glucosyl-8-glucosylgenistein
**2**	8.2	258, 290 sh	593	[MS^2^ 593]: 503 (<1), 341 (5), 311 (100), 283 (7) [MS^3^ 311]: 311 (15), 283 (100), 267 (1)	8-(Glucosyloxyglucosyl)genistein
**3**	8.7	261, 271 sh, 322 sh	639^a^	[MS^2^ 639]: 593 (10), 591 (2), 431 (100), 269 (2) [MS^3^ 431]: 431 (25), 355 (4), 323 (5), 311 (8), 283 (9), 268 (100)	4´,7-Di-*O*-glucosylgenistein
**4**	9.2	272, 293, 301, 314	593	[MS^3^ 593]: 575 (8), 503 (30), 473 (100), 455 (4), 383 (22), 353 (39) [MS^3^ 473]: 455 (2), 383 (17), 353 (100)	6,8-Diglucosylapigenin
**5**	9.6	–	563	–	Unknown
**6**	10.5	261, 290 sh	563	[MS^2^ 563]: 341 (6), 311 (100), 283 (11) [MS^3^ 311]: 311 (100), 283 (39)	8-[Apiosyl-(1 → 6)-glucosyl]genistein
**7**	12.2	240, 263, 322	609	[MS^2^ 609]: 447 (100), 285 (6) [MS^3^ 447]: 447 (2), 285 (100)	3′,7-Di-*O*-glucosylluteolin (0.6758, PR100804)
**8**	13.6	241, 257, 294, 350	609	[MS^2^ 609]: 447(2), 343 (8), 301 (100), 271 (8), 255 (4), 179 (3) [MS^3^ 301]: 301 (100), 271 (42), 255 (28), 239 (3), 229 (3), 211 (1), 179 (44)	Rutin (0.4606, PR100804)
**9**	15.1	239, 257, 293, 348	623	[MS^2^ 623]: 315 (100), 300 (19), 271 (5), 255 (3) [MS^3^ 315]: 315 (90), 300 (100), 287 (5), 272 (2), 271 (2)	3-*O*-Rutinosylisorhamnetin (0.4609, PR100658)

*sh* shoulder

^a^This compound ionized as [M + HCOO]^−^

In conclusion, *Genista tenera n*-butanol extract showed a significant inhibition of glucose-6-phosphatase and alpha-glucosidase enzymes, which denotes a physiologically important role in the metabolism of carbohydrates, and suggests a possible mechanism of action for its antihyperglycemic activity. Moreover, the anti-inflammatory capacity of the extract, and its protective effect against UV-radiation injury are reliable properties that can be attributed to flavonoid diglycosides, the main components of the extract. These components are able to pass the gastrointestinal tract, maintaining their chemical structure and antioxidant activity. The present work adds further support to the previously reported bioactivities of flavonoids from *G. tenera* extracts and contributes to the valorisation of this plant as a source of bioactive compounds that can be regarded as potential prototypes for the generation of new antidiabetic agents with anti-inflammatory and antioxidant properties.

## Experimental

### Chemicals and Reagents

The following reagents used for the evaluation of *α*-glucosidase and glucose-6-phosphatase inhibition were purchased from Sigma (St. Louis, MO, USA): rat intestine acetonic fraction, 0.1 M maleate buffer (pH 6.9), maltose, diagnostic kit for the determination of glucose (GO), acarbose, glucose-6-phosphate, glucose-6-phosphatase, HEPES (pH 6.5), EDTA, DMSO, and phlorizin.

To evaluate cell viability, the reagents MTT [3-(4,5-dimethylthiazol-2-yl)-2,5-diphenyltetrazolium bromide], dimethyl sulfoxide (DMSO), minimum essential medium (MEM), foetal calf serum (FCS), trypsin, phosphate buffered saline(PBS), amphotericin B, penicillin and streptomycin were purchased from Sigma (St. Louis, MO, USA). The H-4-II-E (rat hepatoma) cells were purchased from the European Collection of Cell Cultures.

Cyclooxygenase 1 from sheep (Sigma-Aldrich, Barcelona, Spain) was used to evaluate the anti-inflammatory activity. For the HPLC analysis of the extracts, HPLC-grade methanol and trifluoroacetic acid were purchased from Merck (Darmstadt, Germany). For the antioxidant activity and in vitro digestions with gastric and pancreatic artificial juices, 2,2-diphenyl-1-picrylhydrazyl (DPPH), pepsin and pancreatin were obtained from Sigma-Aldrich (Barcelona, Spain), while potassium phosphate buffer salts were purchased from Merck (Darmstadt, Germany). All reagents used in the present work were of analytical grade.

### Plant Identification and Extract Preparation

The plant was collected in Porto Novo, Island of Madeira, and identified by Dr. Roberto Jardim. A voucher specimen (MADJ 10673) is deposited in the Herbarium of Jardim Botânico da Madeira, Funchal, Portugal.

The powdered plant aerial parts (487.30 g) were exhaustively extracted with ethanol (3 × 2.5 L) at room temperature. The ethanol solution was filtered (Whatman no. 4) and concentrated to dryness in a rotary evaporator at 40 °C and low pressure, giving a crude extract (62.19 g) to which hot (80–90 °C) distilled water (500 mL) was added. The aqueous fraction was then filtered and extracted sequentially by partition with diethyl ether (3 × 500 mL), ethyl acetate (3 × 500 mL) and *n*-butanol (3 × 500 mL). Solvents were evaporated under vacuum to give the diethyl ether (1.23 g), ethyl acetate (5.19 g), and *n*-butanol (5.16 g) dried extracts.

### Enzyme Inhibition Assays

#### *α*-Glucosidase Inhibition

Enzyme solutions were prepared using rat intestinal acetone powder as the source of α-glucosidase. Rat intestinal acetone powder (50 mg) was homogenized with maleate buffer 0.1 M, pH 6.9 (10 mL) and centrifuged at 6000×*g* for 20 min at 4 °C. The supernatant obtained was used as the enzyme solution for the α-glucosidase reaction. Before starting the test, extract samples were dissolved in dimethyl sulfoxide (DMSO) at a concentration of 20 mg/mL. Then, each sample (10 µL) was diluted with maleate buffer 0.1 M, pH 6.9 (90 µL). Experimental procedure for α-glucosidase inhibition was conducted according to the method of Mai et al. [11] with some modifications. Enzyme solution (50 µL) was pre-incubated with the extract solution (50 µL) and maleate buffer 0.1 M, pH 6.9 (100 µL) at 37 °C for 10 min, and then the enzyme reaction was started by adding maltose substrate solution (50 µL, 1 % w/v in maleate buffer 0.1 M, pH 6.9). The enzymatic reaction was allowed to proceed at 37 °C for 10 min, and then stopped by heating at 100 °C for 5 min. The reaction mixture was kept in ice bath. The generated glucose was measured with a commercial glucose assay kit (GO) at 540 nm, in a Hitachi U-2000 spectrophotometer. A negative control was run with maleate buffer 0.1 M, pH 6.9 (150 µL) in the absence of the extract. For blank determination, the enzyme solution was replaced by maleate buffer 0.1 M, pH 6.9 (50 µL), and the same procedure as described above was carried out. Acarbose was used as a reference compound and tested at the some concentration of the extract (400 mg/L). A total of five separate trials were carried out. The α-glucosidase activity is expressed in percentage of the activity of the free enzyme.

#### Glucose-6-Phosphatase Inhibition

Enzyme solution was prepared with rabbit liver microsomes as a source of glucose-6-phosphatase. This solution was prepared according to the method of Marccuci et al. [26] cited by Estrada et al. [13]. Briefly, microsomal protein (16.74 mg) was homogenized with HEPES 5 mM, pH 6.5 (0.837 mL), sucrose (0.25 mM) and MgCl_2_ (1 mM) and frozen at −80 °C until use. Before starting the test, extract samples were dissolved in dimethyl sulfoxide (DMSO) at a concentration of 20 mg/mL. Then, each sample (40 µL) was diluted with HEPES 16 mM, pH 6.5 (60 µL). Experimental procedure for glucose-6-phosphatase was conducted according to the method described in Estrada et al. [13], with some modifications. In brief, phlorizin (5.5 µL) and extracts (5 µL) were previously incubated with the enzyme (2.5 µL) at 37 °C for 40 min and G-6-P solution was added to start the reaction. The glucose-6-phosphatase assay was carried out in a final volume of 100 µL, with glucose-6-phosphate (5 mM), EDTA (2 mM), HEPES, pH 6.5 (16 mM) and the enzyme mixture. The reaction was carried out at 37 °C for 60 min, without shaking, and was stopped by the addition of 1 mL of ammonium molybdate (0.28 %), SDS (1.11 %) and ascorbic acid (1.11 %) in sulphuric acid (0.33 M). The reaction was incubated at 47 °C for 20 min and the absorption at 820 nm was recorded in a Hitachi U-2000 spectrophotometer. The final concentration of DMSO in experimental assays was 2 %. Phlorizin was dissolved in DMSO (10 %) and used as positive control in the same concentration as the extracts (400 mg/L). A total of twelve separate trials was carried out. The glucose-6-phosphatase activity is expressed in percentage of the activity of the free enzyme.

#### COX-1 Inhibition

COX-1 (EC 1.14.99.1) activity was measured by following spectrophotometrically at 611 nm the oxidation of TMPD with arachidonic acid. This assay was performed as described in [27], with some modifications. Briefly, to Tris–HCl buffer 100 mM, pH 8 (1000 µL) COX-1 enzyme (200 units dissolved in Tris–HCl buffer, 50 µL) was added and the co-factor of hematin dissolved in Tris–HCl buffer (100 µL, 3 µM in the assay). After 5 min, the enzyme was reconstituted and then a solution of plant extract or a standard inhibitor in DMSO (50 µL) and pure DMSO (50 µL) were added and pre-incubated for 3 min at 25 °C. After the incubation time has expired, TMPD dissolved in Tris–HCl buffer (200 µL, 100 µM in the assay) was added. The reaction was initiated by the addition of arachidonic acid dissolved in DMSO (50 µL, 100 µM in the assay) to the enzyme/extract mixture and contents were mixed immediately. The initial velocity of the reaction was measured following the oxidation reaction of TMPD at 611 nm for 30 s. The inhibition % was calculated as follows:

$$I\,(\% )\, = \,100\, - \,\left( {{{V_{i\,with\,inhibitor} } \mathord{\left/ {\vphantom {{V_{i\,with\,inhibitor} } {V_{i\,with\,out\,inhibitor} }}} \right. \kern-0pt} {V_{i\,with\,out\,inhibitor} }}} \right) \times \,100$$

where *V*_*i* with inhibitor_ is the initial velocity observed at different inhibitor concentrations and *V*_*i* without inhibitor_ is the velocity observed for enzyme samples pre-incubated for the same time with inhibitor free in DMSO. IC_50_ values were determined by regression analysis.

### Cell Viability Assay

Cell viability was studied by the MTT [3-(4,5-dimethylthiazol-2-yl)-2,5-diphenyltetrazolium bromide] method. The test described below is adapted to be used with cell cultures in growth phase.

#### Cell Culture

The H-4-II-E cells were seeded into tissue culture flasks of 75 cm^2^ in MEM medium supplemented with 10 % FCS and 1 % antibiotics containing penicillin (10000 U/mL) and streptomycin (10 mg/mL) and also 0.1 % antifungal, amphotericin B (250 mg/mL). The cells have multiplied freely to 80–90 % confluence at 37 °C in a humidified atmosphere of 10 % CO_2_ (incubation chamber Shel Lab CO_2_ Series). The medium was changed every 3 days. When the cultures were 80–90 % confluent, H-4-II-E cells were washed with PBS (10 mL), detached with trypsin (1 mL), counted and suspended in MEM medium to be seeded in 96-well plates at a density of 5 × 10^4^ cells/mL. Assays were performed in a laminar flow camera with biosafety class II A/B3 (Steril Gard–Baker Company). Before starting the test, samples were dissolved in DMSO, at a concentration of 20 mg/mL, and at least five separate trials were made.

#### MTT Assay

MTT assay was performed according to INVITTOX Method N. 17 for basal cytotoxicity (ECVAM) [28]. Exponentially growing cells (H-4-II-E) were trypsinized and suspended in fresh media (MEM supplemented with 10 % fetal bovine serum and 100 U/mL penicillin, and 100 mg/mL streptomycin) at a density of 5 × 10^4^ cells/mL, and this cell suspension (100 µL) was plated on to each well of a 96-well plate. Cells were incubated at 37 °C, 5 % CO_2_ humidified incubator for 24 h, after which the *n*-butanol extract and ascorbic acid (400 mg/L), the positive control, and DMSO, the negative control, were added. The cells were placed in the incubator for 1 h and then, half of the plates were exposed to UV-C radiation (λ = 257.7 nm, Philips TUV G15T8 UVC lamp) for 5 min. Cells were further incubated until a total of 48 h and then MTT (10 μL, 10 mg/mL) was added to each well. After 4 h incubation at 37 °C the medium was removed and the wells were washed with cold (5 °C) PBS (100 μL).The formazan crystals were solubilized by adding DMSO (100 μL) to each well and placing the plates in an orbital shaker for 20 min. Colorimetric reading was done in a microplate reader (Beijing Perlong New Technology DNM-9602) at 492 and 630 nm (reference λ). The viability is expressed as an average percentage of metabolically viable cells in each group compared to control cells. Results are averages from five separate assays.

### In Vitro Digestion

The gastrointestinal digestion of the plant extract was simulated in vitro with artificial gastric and pancreatic juices using the method described by Falé et al. [19], and the chemical composition of the digested extract was analysed by HPLC–DAD while the remaining antioxidant activity was measured by the DPPH method.

#### In Vitro Metabolism by the Gastric Juice

Gastric juice (2.5 mL) was added to the extract solution (5 mg/mL) in water (2.5 mL). The mixture was incubated at 37 °C for 4 h. Samples (100 µL) were taken hourly, added to ice-cold methanol (900 µL) and analysed by HPLC. The gastric juice (100 mL) consisted of pepsin (320 mg) and NaCl (200 mg), and the pH was set to 1.2 with concentrated HCl. Assays were done in triplicate.

#### In Vitro Metabolism by the Pancreatic Juice

Pancreatic juice (2.5 mL) was added to the extract solution (10 mg/mL) in water (2.5 mL). The mixture was incubated at 37 °C for 4 h. Samples (100 µL) were taken hourly, added to ice-cold methanol (900 µL) and centrifuged for 5 min at 5000×*g*. The supernatant was analysed by HPLC. The pancreatic juice consisted of pancreatin (250 mg) in potassium-phosphate buffer 50 mM, pH 8 (10 mL). Assays were done in triplicate. Samples (200 µL) were taken at the same time, centrifuged 5 min at 5000×*g* and the supernatant was analysed for antioxidant activity, against a blank with water instead of plant extract. Assays were done in triplicate and the concentration of the extract in the digestions varied according to the IC_50_ values for antioxidant activity.

### Antioxidant Activity

Antioxidant activity was evaluated by the DPPH method. To a solution of DPPH (2.5 mL, 0.002 % in methanol), 25 µL of plant extract (100–300 µg/mL in the cuvette) were added. The mixture was incubated for 30 min at room temperature. The absorbance was measured at 517 nm against a corresponding blank. The antioxidant activity was calculated as follows:

$${\text{AA }}\left( \% \right) = \left( {{\text{A}}_{\text{DPPH}} - {\text{A}}_{\text{Sample}} /{\text{A}}_{\text{DPPH}} } \right) \times 100$$

where AA is the antioxidant activity, A_DPPH_ is the absorption of the DPPH solution against the blank, A_Sample_ is the absorption of the sample against the blank. The tests were carried out in triplicate and the extract concentration providing 50 % of antioxidant activity (IC_50_) was obtained by regression analysis.

### Phytochemical Analysis

HPLC analysis was carried out in an Elite LaChrom^®^ VWR Hitachi Liquid Chromatograph equipped with a Column Oven L-2300 and Diode Array Detector L-2455 (VWR, USA). A column LiChroCART^®^ 250-4 LiChrospher^®^ 100 RP-8 (5 µm) from Merck (Darmstadt, Germany) was used. The extract was analysed by injecting the sample (25 µL, 1 mg/mL) with an auto injector, and using a gradient composed of solution A (0.05 % trifluoroacetic acid), and solution B (methanol) as follows: 0 min, 80 % A, 20 % B; 20 min 20 % A, 80 % B; 25 min, 20 % A, 80 % B. The flow was 1 mL/min, and the detection was carried out between 200 and 500 nm with a diode array detector.

LC–MS and LC–MS^n^ analysis were carried out on a liquid chromatograph Surveyor Plus Modular LC system connected to a LCQ Duo ion trap mass spectrometer equipped with an electrospray ionisation (ESI) source, from Thermo Scientific (Bremen, Germany). The column used was a LiChroCART^®^ 250-4 LiChrospher^®^ 100 RP-8 (5 µm) column (Merck, Darmstadt, Germany). The extract was analysed by injection of the sample (25 µL, 10 mg/mL) and using a linear gradient composed of solution A (1.0 % formic acid), and solution B (methanol) as follows: 0 min, 70 % A, 30 % B; 20 min 20 % A, 80 % B; 25 min, 20 % A, 80 % B. The mass spectrometer was operated in both positive and negative ion modes in the range *m/z* 120–1000 and the parameters were adjusted in order to optimize the signal-to-noise ratios (S/N) for the ions of interest. Briefly, the nebulizing and auxiliary gas (nitrogen) flow rates were 40 and 20 (arbitrary units) and the capillary temperature was set to 250 °C. Collision induced dissociation (CID) experiments were performed by isolating the ions within the ion trap and accelerating them in order to suffer multiple collisions with the background gas present in the ion trap (helium) using a data dependent acquisition mode. The ions of interest were activated by applying a percentage of a supplementary a.c. potential in the range of 0.75–1.75 Vp–p (peak-to-peak) to the end cap electrodes of the ion trap at the resonance frequency of the selected ion (referred to as the normalized collision energy, NCE). The injection times were 50 ms in a full scan and 200 ms in a MS/MS scan. Xcalibur™ software from Thermo Scientific was used to acquire and to process the data.

The identification of some extract constituents was performed using the MassBank database (freely available at http://www.massbank.eu/MassBank/).
